# A survival case of intravenous paraquat intoxication: A case report

**DOI:** 10.1097/MD.0000000000011669

**Published:** 2018-07-27

**Authors:** Chi-Wei Chen, Yen-Hung Wu, Shun-Ching Chien, Jhong-Ching Lin

**Affiliations:** a Department of Emergency Medicine, Kaohsiung Medical University Hospital; b School of Medicine, College of Medicine, Kaohsiung Medical University, Kaohsiung, Taiwan.

**Keywords:** acute kidney injury, hemoperfusion, intravenous paraquat intoxication

## Abstract

**Rationale:**

Paraquat, an agent highly toxic to humans and animals, is a widely used herbicide and also commonly used for suicide attempts in Taiwan. The most common route of intoxication is oral ingestion, and parenteral poisoning is respectively rare.

**Patient concerns:**

A 39-year-old illicit abuser of heroin and amphetamine injected 0.5 mL of 24% paraquat directly into his right cephalic vein due to hallucination. The patient was brought to our emergency department for management 4 hours after injection. He was fully conscious and had normal vital signs. Systemic review showed mild dyspnea, abdominal pain and right wrist pain over the injection site. The only abnormal physical finding was erythema over the injection site and epigastric tenderness.

**Diagnosis:**

Laboratory investigations, including complete blood count, liver and renal function, and electrolytes initially yielded normal results. Urinalysis showed normal findings except a positive urine paraquat test (4+). The initial plasma paraquat concentration was 0.51 μg/mL.

**Interventions:**

He was admitted to the intensive care unit and underwent one session of charcoal hemoperfusion therapy. Acute kidney injury developed on the fourth day after intoxication, with the level of serum creatinine rising rapidly from 0.96 to 4.57 mg/dL and the daily urine output decreased noticeably from > 2000 to 900 mL. The serum creatinine level improved gradually with adequate fluid supplementation.

**Outcomes:**

The patient was discharged 13 days later in a stable condition.

**Lessons:**

Intravenous paraquat intoxication is rare. Patients who suffer from intravenous intoxication may not directly suffer from mucosal irritation, but the clinical onset of systemic effects is more immediate and lethal. The prognosis of paraquat poisoning is determined by the time of poisoning and the plasma paraquat concentration before treatment. Proudfoot's curve provides a simple method of predicting the survival rate. The most effective mode of management is extracorporeal therapy, and immunosuppressive or antioxidant therapies have shown insufficient evidence of benefit.

## 1 Introduction

Paraquat, an agent highly toxic to humans and animals, is a widely used herbicide and also commonly used by people attempting suicide in Taiwan. Paraquat is highly toxic and causes impairment of several vital organs (such as the liver, kidney, and lung) and almost all poisoned individuals die due to multiorgan failure, including profound metabolic acidosis, depression of myocardial or respiration function due to subsequent pulmonary fibrosis, and renal or hepatic failure.^[1]^ The most common route of poisoning is oral ingestion; parenteral poisoning is respectively rare. We report a case that survived after intravenous paraquat injection. After we contacted the regulatory institutional review board of the Kaohsiung Medical University Hospital, ethical approval was not required for this case report article. Informed consent was obtained from the patient for the publication of this case report.

## 2 Case report

The patient was a 39-year-old man and an illicit abuser of heroin and amphetamine. He injected 0.5 mL of 24% paraquat directly into his right cephalic vein due to hallucination. The patient was brought to our emergency department for management 4 hours after injection. He was fully conscious and had normal vital signs (pulse rate of 63 beats/min, respiratory rate of 16 breaths/min, and blood pressure of 112/69 mm Hg), except for mild hypothermia (body temperature of 35.8°C). Systemic review showed mild dyspnea, abdominal pain, and right wrist pain over the injection site. The only abnormal physical finding was the erythematous injection site and epigastric tenderness. Laboratory investigations, including a complete blood count, liver and renal function, and electrolytes initially yielded normal findings. Urinalysis yielded normal results, except the positive urine paraquat test (4+). The initial plasma paraquat concentration was 0.51 μg/mL. A chest radiograph was also showed normal findings. He was admitted to the intensive care unit and underwent one session of charcoal hemoperfusion therapy. A follow-up urine paraquat test performed 2 days later yielded negative results. He did not receive methylprednisolone or cyclophosphamide therapy. Acute kidney injury developed on the fourth day after intoxication, with the level of serum creatinine rising rapidly from 0.96 to 4.57 mg/dL and the daily urine output noticeably decreasing from > 2000 to 900 mL. We administered adequate fluid supplementation, keeping balance of urine output, and avoiding nephrotoxicity medication. The serum creatinine level improved gradually. Intermittent postprandial abdominal pain and constipation were found after paraquat poisoning. Otherwise, there was no dyspnea or other discomfort during hospitalization. The patient was discharged 13 days later in a stable condition.

## 3 Discussion

Paraquat intoxication is a common cause of fatal herbicide intoxication. Various routes of intoxication have been reported, with the most common being unintentional and intentional oral ingestion, skin, or respiratory tract; however, the parenteral route has rarely been reported (Table 1).^[1–7]^

**Table 1:**
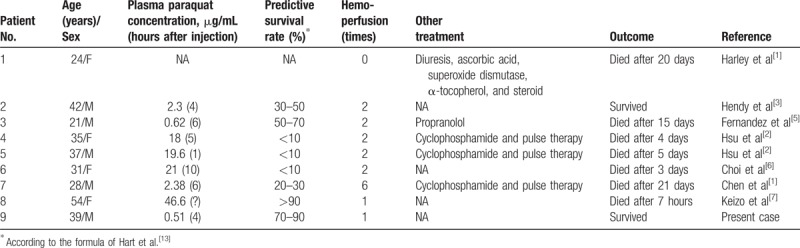
Summary of management and outcome of patients with parental paraquat intoxication.

The presentations of paraquat intoxication include local damage and systemic toxicological effects. The local symptoms are directly related to mucosal damage, such as ulceration, hemorrhage or even perforation of the gastrointestinal (GI) tract, and other GI tract irritation symptoms like nausea, vomiting, and diarrhea. Patients who inject paraquat intravenously will experience local skin or vascular symptoms. In previously reported cases, patients with intravenous paraquat poisoning experienced some GI symptoms, such as nausea or vomiting.^[2,3,6]^ Our patient suffered from intermittent abdominal pain or chronic constipation, which may be explained by the systemic effect of paraquat on the central nervous system.^[2,8]^

The systemic effects, such as renal, hepatic, or pulmonary damage, occur later than local effects, and are suspected to be dose dependent and lethal. Our patient suffered from acute kidney injury without other obvious hepatic or pulmonary damage. This might be due to the small dose injected and early initiation of hemoperfusion.

There is still no definitive treatment for paraquat intoxication, but previous studies suggested therapies including prevention of absorption from the GI tract, increasing the elimination from plasma, and preventing further pulmonary damage. Patients who inject paraquat intravenously do not require GI decontamination such as the use of activated charcoal. Extracorporeal therapy, such as hemoperfusion, is suggested to be initiated within 4 hours after exposure, and should be repeated daily until the urine or serum paraquat test yield negative results.^[8]^ In preventing pulmonary damage, several methods were proposed, including anti-inflammatory and immunosuppressive therapy, antioxidant therapy, and avoiding oxygen therapy unless there is marked hypoxia.^[8]^ The benefit of immunosuppressive therapies with glucocorticoid and cyclophosphamide are still controversial in several randomized controlled or uncontrolled trials^[9,10]^ and meta-analysis.^[11]^ Several antioxidants, including *N*-acetylcysteine, deferoxamine, vitamin E, and vitamin C, have been suggested to prevent pulmonary damage, but there is insufficient evidence of their benefit. In our case, the patient received hemoperfusion only, without glucocorticoid and cyclophosphamide therapy. Because the effect of glucocorticoid and cyclophosphamide is still controversial.

According to the paraquat concentration-time ratio proposed by Proudfoot et al in 1979, relatively high survival rates were suggested if the concentrations of serum paraquat do not exceed 2.0, 0.6, 0.3, 0.16, and 0.1 mg/L at 4, 6, 10, 16, and 24 hours.^[6,12]^ Our patient's initial plasma paraquat concentration was 0.51 μg/mL, which reflected a 70% to 90% survival rate according to Proudfoot's curve.^[13]^

## 4 Conclusion

Intravenous paraquat intoxication is still rare. Patients who experience intravenous intoxication may not directly suffer from mucosal irritation, but the clinical onset of the systemic effects are more immediate and lethal. The prognosis of paraquat poisoning is determined by the time of poisoning and plasma paraquat concentration before treatment. Proudfoot's curve provides a simple method of prediction of the survival rate. The most effective mode of management is extracorporeal therapy, and immunosuppressive or antioxidant therapies have shown insufficient evidence of benefit.

## Author contributions

**Resources:** Shun-Ching Chien.

**Supervision:** Jhong-Ching Lin.

**Visualization:** Yen-Hung Wu.

**Writing – original draft:** Chi-Wei Chen.

**Writing – review & editing:** Chi-Wei Chen, Yen-Hung Wu, Jhong-Ching Lin.
